# “We can all relate”: patient experience of an emotion-oriented group intervention after Acquired Brain Injury

**DOI:** 10.3389/fpsyg.2024.1384080

**Published:** 2024-06-27

**Authors:** Leanne Rowlands, Christian Salas, Rudi Coetzer, Sharon Buckland, Oliver H. Turnbull

**Affiliations:** ^1^School of Psychology, Arden University, Coventry, United Kingdom; ^2^Department of Psychology, Bangor University, Bangor, United Kingdom; ^3^Clinical Neuropsychology Unit, Centre for Human Neuroscience and Neuropsychology, Faculty of Psychology, Diego Portales University, Santiago, Chile; ^4^Brainkind, Sussex, United Kingdom; ^5^Medicine, Health & Life Science Faculty, Swansea University, Swansea, United Kingdom; ^6^North Wales Brain Injury Service, Betsi Cadwaladr University Health Board, Colwyn Bay, United Kingdom

**Keywords:** brain injury, groups, neurorehabilitation, social, emotion regulation, qualitative

## Abstract

**Introduction:**

Group interventions are carried out routinely across neuropsychological rehabilitation services, to improve understanding of brain injury and aspects of impairment. Treatment provided in a group modality can bring additional perceived benefits, such as co-operative learning. However, there are very few studies which explore patient perceptions and experiences of such interventions. In the present study we investigated the experience of attending a group-based educational intervention for the consequences of acquired brain injury (ABI), which had a strong focus on emotion and emotion regulation.

**Methods:**

Using qualitative semi-structured interviews (approximately 20 minutes), the study explores the lived experience of participating in the seven-session programme, the better to identify the perceived efficacy, salience and value of individual elements. Twenty participants with ABI took part in individual interviews, after completion of the group programme (the Brain Injury Solutions and Emotions Programme, BISEP). The study adopted a descriptive phenomenological philosophy, which focuses on lived experience to explore a phenomenon (i.e. the experience of BISEP). As regards methods, the study employed thematic analysis to cluster experiences into themes of meaning.

**Results:**

Five themes were identified: (1) ‘Long term consequences and psychological needs’, which related to the persistent nature of direct consequences of injury and adjustment, and how these result in a need for interventions such as BISEP. (2) ‘Positive experiences of participating in the programme’, referred to participants’ overall experience of the programme and valued elements within it. The remaining themes referred to the programme as (3) a social milieu; (4) a place to learn; and (5) a place to promote positive emotional experiences.

**Discussion:**

Similar to previous studies, many people reported high acceptability and perceived value of the group programme, and its role in facilitating adjustment and understanding of injury. Of particular importance was the opportunity to socialise with people who “can all relate”, in line with a growing emphasis on social rehabilitation. The findings especially highlight the relevance of emotion-focused group programmes for ABI, promoting emotion regulation, and practical tools that are delivered optimistically. Further implications for practice and future research include to focus on long term rehabilitation, a social milieu, and strategies to support adjustment.

## Introduction

Neuropsychological rehabilitation has increasingly been focusing on adjustment and acceptance, consistent with an ‘emotional turn’, where feelings are placed at the heart of formulation (Wilson et al., 2009; Wilson and Gracey, 2009; Bowen et al., 2010; McDonald, 2017). Group interventions are promising vehicles to promote understanding of injury, psychological adjustment, and improve aspects of impairment (Psaila and Gracey, 2009; Patterson et al., 2016; Wilson, 2017). Evidence points to their efficacy for a range of targeted outcomes, such as cognitive impairment and coping skills (Backhaus et al., 2010; Patterson et al., 2016, for a review). However, there has been only modest *qualitative* research capturing the perceptions and experiences of patients to inform group intervention development and evaluation (Patterson et al., 2016).

Within neuropsychological rehabilitation, *holistic* approaches have received much attention in the literature (see Ben-Yishay, 2000; Ben-Yishay and Daniels-Zide, 2000; Trexler, 2000; Wilson et al., 2009; Ben-Yishay and Diller, 2011), and are effective (Cicerone et al., 2008; Turner-Stokes et al., 2015). Traditionally, holistic approaches involve very intense provision of individual and group interventions, to increase understanding and self-awareness, and address the cognitive, behavioral, and emotional consequences of ABI as a whole, providing compensation strategies to help manage difficulties (Ben-Yishay, 2000; Trexler, 2000; Wilson et al., 2009). Despite their efficacy, the service intensity and the high staff-to-patient ratio means that holistic programs are often expensive and time-limited.

In recognition that subjective experiences play an important role in recovery, contemporary holistic approaches also address embodied perspectives of consciousness (see Lo et al., 2023 for review). Embodiment in this context refers to how survivors experience and perceive the world through their ‘lived body’ – including emotions, sensations, perceptions, and all aspects of self-awareness within a physical body (Merleau-Ponty, 1962; Lo et al., 2023). A phenomenologically-informed neurorehabilitation approach considers individual experience, the body, and the entire surrounding environment relevant for recovery. For example, rehabilitation of gait disturbances in a music environment for people with Parkinson’s disease has shown promising motor, cognitive, and socio-affective outcomes (Schiavio and Altenmüller, 2015), and a case study of a woman with ABI receiving rehabilitation on a therapy ball (as opposed to a chair) demonstrating greater cognitive and behavioral performances (Martínez-Pernía et al., 2016). See Martínez-Pernía (2020) for a detailed account of experiential neurorehabilitation based on an embodied perspective. Despite its clinical relevance, such an experiential approach to rehabilitation has not yet widely transformed standard practice.

Due to the chronic nature of brain injury, survivors can experience difficulties for many years, highlighting the *continued* role of rehabilitation in the community (Coetzer, 2008; Turner-Stokes et al., 2015). However, the provision of neuropsychological rehabilitation in the chronic phase has been an area of little emphasis in the wider field. There are some reports that holistic approaches can be adapted to overcome barriers in low resource and long-term community settings (e.g., Coetzer, 2008; See Balchin et al., 2017, for a handbook on this topic). The North Wales Brain Injury Service (NWBIS), UK, one program which follows the philosophical foundations of the holistic model, have published extensively on this topic. NWBIS provides rehabilitation in a long-term, ‘slow stream’, out-patient setting, and provides individual rehabilitation and *group* interventions (Coetzer et al., 2003; Coetzer, 2008; Coetzer et al., 2018). Group-based treatment in such services typically consist of several weekly sessions, run over the course of three-weeks to three months (Patterson et al., 2016). Psycho-education about brain injury, compensation strategies for cognitive difficulties, and facilitation of emotional adjustment and awareness, are provided holistically within one program.

Interventions are carried out routinely in groups across rehabilitation settings (Tyerman and Hucker, 2016). Many, however, adopt a ‘home-grown’ approach, where interventions have been developed by clinicians at services, but are typically not evaluated empirically. Additionally, several published guides can be used to facilitate group programs (e.g., Van den Broek and Dayus, 2002; Powell, 2013; Winson et al., 2017), however these are not always evidence-based and do not have associated empirical data. The existing evidence-base for *empirically-evaluated* group interventions is complex, with large variation in their targeted outcome, setting and duration of delivery, intervention content, and methodological rigor (Patterson et al., 2016). Considered together, quantitative evidence suggests that group-based treatment is an effective intervention approach (Patterson et al., 2016), however further research is required to establish a more robust evidence-base.

The majority of evaluated group interventions have focused on cognitive difficulties (Patterson et al., 2016). Some have also focused on adjustment and coping (e.g., Bradbury et al., 2008; Backhaus et al., 2010), and group programs with a multidisciplinary focus (e.g., Malec, 2001). In addition to targeted outcomes, there are other benefits to group environments in ABI rehabilitation (Winson et al., 2017, p. 9). For instance, they provide an opportunity for co-operative learning, and are valued by patients and carers (Couchman et al., 2014). Providing *group* rehabilitation also simultaneously provides an opportunity for increasing self-awareness and social support (Anson and Ponsford, 2006; Lundqvist et al., 2010), and developing social connections with other survivors to help fight social isolation after ABI (Salas et al., 2018). There are, however, few evaluations of group programs which consider multiple aspects of ABI consequences, with an underlying holistic philosophical approach. More research is, therefore, necessary to ensure that such interventions are acceptable, efficacious, and an appropriate use of limited service resources.

A scoping review of group interventions in neuropsychological rehabilitation especially noted an absence of *qualitative* research, that consider participants’ perceptions of group processes and elements of intervention (Patterson et al., 2016). Understanding the ‘user experience’, or participants’ subjective accounts, in the delivery of neuropsychological interventions are key to develop and improve group programs. *Qualitative* research has been crucial in developing an understanding of the wider context of the rehabilitation experience (Wain et al., 2008; Levack et al., 2010; Graff et al., 2018). One patient’s account captured the discrepancy between a lack of improvement on empirical measurements and the feelings of having improved (Wain et al., 2008). This indicates the value and clinical relevance of qualitative patient accounts and experiences in refining services and interventions.

One aspect of ABI consequences that seems particularly important to include in group programs is emotion regulation (ER). This is because difficulties with emotion management, or *emotion dysregulation*, may be a key transdiagnostic factor of emotional difficulties after ABI (Shields et al., 2016), and have been identified as a *common* consequence of focal and diffuse brain injury (Bechara, 2004; Beer and Lombardo, 2007; Obonsawin et al., 2007). ER refers to processes which can influence emotion type, their intensity, and how they are experienced and expressed (Gross and Thompson, 2007). The most popular model of ER, the Process Model, includes five ER strategies, that can be implemented at key time points (Gross and Thompson, 2007; Gross, 2013). These are: (1) *situation selection*: choosing settings which give rise to desirable, or undesirable, emotions *before* the event; (2) *situation modification:* taking steps which change the external environment to alter the emotional impact of the situation; (3) *attentional deployment*: changing attentional focus, often by focusing on more desirable internal scenarios, (4) *cognitive change (reappraisal):* changing the meaning of a situation to alter its emotional impact, (5) *response modulation:* altering emotional response tendencies once they have been elicited (Gross and Thompson, 2007; Werner and Gross, 2010; Gross, 2015).

Many group interventions include an *element* of ER (Patterson et al., 2016, for review; Winson et al., 2017), including interventions with a Cognitive Behavior Therapy (CBT) focus (e.g., Bradbury et al., 2008; Aboulafia-Brakha et al., 2013). However, there is little consistency in the operational definitions of ER, and CBT-based interventions require appropriately trained facilitators. The available studies of targeted ER interventions appear to be growing, but still remain sparse, and with much variation in their theoretical approaches and success (Cantor et al., 2014; Tornås et al., 2016). An example of a successful ER group program is that developed by Tsaousides et al. (2017). This 24-session, web-based, group intervention conceptualized ER based on the Process Model, and involved training on specific ER strategies. Significant and continued improvement was found on the primary outcome measure of ER difficulties. However, publicly-funded services may not have the resources to carry out 24-sessions of video-conferencing, or allocate clinician time to run interventions that focus *exclusively* on ER. A potential avenue for such settings would be to incorporate a theoretically sound framework of ER in holistic education-based group interventions, alongside the traditional topics that are commonly seen in such programs.

Little is known about patients’ experiences of ER training provided in a group format. There are, however, a few exceptions to this. For example, Tsaousides et al. (2017) included very brief qualitative feedback interviews in their online ER intervention, and found that participants considered the program to be relevant, and enjoyed connecting with other survivors. This is an important finding, because both *experience* and *engagement* in rehabilitation programs are indicated as crucial components for outcomes (Paolucci et al., 2012; Williams et al., 2019). Though it is an individualized intervention, Karagiorgou et al. (2018) noted how Positive Psychotherapy, and specific components such as making a note of ‘Three Good Things’ in a day, facilitated positive personal growth. Due to the distressing nature of emotional difficulties after ABI (Ownsworth and Fleming, 2005; Levack et al., 2010), it is especially important to address these issues in interventions, and understand the experience and meaning of participating in such group programs and their perceived role in outcomes.

Qualitative accounts have proven important in developing our understanding of which elements are important for various rehabilitation outcomes, from patients’ perspectives (Couchman et al., 2014; Graff et al., 2018). This includes which perceived factors or experiences have positive or negative effects on motivation for rehabilitation, for example adequate provision of information, versus a lack of information or transparency from professionals (Maclean et al., 2000; Graff et al., 2018). In a meta-synthesis of qualitative research, external support (such as that provided by rehabilitation programs) was considered especially important for recovery (Levack et al., 2010). Specifically, survivors discussed the value of learning about the injury, and the ‘normalising’ effect of interacting with other survivors in community settings (Levack et al., 2010).

A number of interesting themes were identified, following the qualitative analysis of a multifamily group therapy (Couchman et al., 2014). The attendees expressed a sense of social connection, the enhancement of a sense of self-identity, and the knowledge and understanding which came from the interactions with other members. This is important, because it highlights the value of the *group* environment, and how the informal learning which comes from it was seen as more informative than the content of the intervention. Giving patients an opportunity to participate in rehabilitation activities with other survivors may bring therapeutic gains in terms of support and guidance, social interaction, and engagement (Patterson et al., 2016, for a review).

From a phenomenological standpoint, Martin et al. (2015) interviewed survivors of ABI to understand their lived experiences of life goals in inpatient neurorehabilitation. The authors acknowledge the importance of exploring the voices and perceptions of those with lived experience as a route to challenge our assumptions of and understand person-centered rehabilitation. For instance, they found that participants felt that rehabilitation targeted instrumental activities, but they desired social connectedness as a central life goal. An important clinical consideration developed from such a phenomenological approach is the need to consider the milieu and strategies to support social connection (Martin et al., 2015). Similarly, Panday et al. (2022) investigated patient perspectives within rehabilitation from a phenomenological philosophy, and found that autonomy, adjustment, and identity are important elements for recovery.

Phenomenology is a philosophy, methodology, and method; and refers to a subjective dimension, a focus on participants’ lived experiences of a phenomenon, and interprets an individual’s consciousness (Giorgi and Giorgi, 2003; Christensen et al., 2017; Gill, 2020; Sinfield et al., 2023) There is a variation of phenomenology types (e.g., transcendental, embodied), but Husserl’s descriptive phenomenology is involved with the description of lived experiences as a representation of a phenomenon and maintains no priori assumptions or deductive logic procedures (Willis et al., 2016). Descriptive phenomenology remains close to participants’ original experiences and meanings to understand phenomena, and thus does not seek to *interpret* meanings (c.f. interpretive phenomenology). Given this closeness to lived experience and people’s voices, descriptive phenomenology has been especially insightful across a range of person-centered healthcare research (e.g., Greenfield and Jensen, 2012; Shorey and Ng, 2022 for reviews). A grounding in descriptive phenomenology as an epistemological framework can lend itself to a range of analysis approaches, including thematizing meaning via thematic analysis (Sundler et al., 2019 for detailed guide).

There are three novel elements in the present study. The first is using descriptive phenomenology as a guiding philosophy to understand the lived experience of participating in a holistically-influenced group program in a low-intensity, long-term, community rehabilitation setting (i.e., the phenomenon of interest). Second, is the focus on participants’ experiences as a route to identify valued elements, perceived efficacy (or inefficacy) and acceptability (or unacceptability) of the program. Third, is to understand participants’ experience of the emotion and emotion-regulation focus of the intervention (which was based upon the over-arching theoretical Process Model [Gross and Thompson, 2007; Gross, 2013]). The present study aimed to describe participants’ lived experience of a newly developed education and skills-based group intervention (The Brain Injury Emotion and Solutions Program [BISEP]), which emphasized ER, alongside several aspects ABI consequences and psycho-education provided holistically.

## Methods

### Design and data analysis

The Standards for Reporting Qualitative Research (SRQR) proposed by O’Brien et al. (2014) are followed. The present qualitative study was underpinned and guided by a descriptive phenomenological philosophy, meaning that our epistemological position is that an individual’s reality (or ‘lifeworld’) is experienced through their consciousness, and we recognize the challenges that come with a focus on an objective reality. We chose this as a guiding philosophy because it was appropriate to address the risk of bias in the present study, due to the group facilitator also conducting the interviews and analysing the data. In line with this philosophy, the researcher’s goal is transcendental subjectivity – that is, the researcher’s biases are assessed through ‘bracketing’ (see Gearing, 2004) and reduction. Phenomenological reduction involves keeping at bay one’s pre-assumptions and theories, and instead stays close to the described phenomenon – this was especially important in the context that the researcher/interviewer was familiar with each individual. Bracketing involves setting aside certain elements (e.g., internal suppositions). We endeavored to set aside our knowledge of the participants’, our perceptions of how they interacted with the program, and any theories and knowledge regarding group interventions, with a pragmatic bracketing approach. A loose pragmatic approach to bracketing was employed, as some argue that completely putting aside assumptions is not possible and some assumptions are part of understanding (see Sundler et al., 2019, for review). A descriptive philosophy underpinning was also chosen because of its emphasis on ‘the lifeworld’ – the lived experience of participants in the present study must be interpreted in line with their subjectivity, interactions, and the ‘world’ of the phenomenon of interest (the group program and the physical and emotional space in which it happens).

Descriptive phenomenology was used to describe and articulate the essential features of the phenomenon of interest (experiences of and during the group rehabilitation program). We let the principles of openness, reflexivity, and questioning pre-understanding guide the entire process (i.e., following suggestions of thematic analysis for descriptive phenomenology by Sundler et al., 2019). The data was then analyzed using thematic analysis (Braun and Clarke, 2006; Maguire and Delahunt, 2017). Thematic analysis is considered an appropriate method for a variety of qualitative research questions, across a range of epistemologies (Nowell et al., 2017). We follow the thematic analysis for descriptive phenomenology guide by Sundler et al. (2019), which follows and inductive approach which is grounded in the data and experience of participants, and involved searching for patterns of meanings and their complexity (not necessarily their frequency) and how these can be organized into themes.

Interviews were conducted in person, and audio-recorded on a Dictaphone. The recording was immediately transferred to a password protected computer after the session, and deleted from the device. After transcribing the interviews *verbatim*, the first step was data familiarization. This was done by the first (LR) and second author (CS) reading and re-reading the transcripts in an open-minded manner to avoid confirmation bias when familiarizing with the data. Next, the transcripts were exported into an Excel spreadsheet. Preliminary codes were generated to identify the essence of the data or meanings, in three waves. Fifty percent of the data was double coded by two independent coders (LR and CS), who first coded one interview before meeting to discuss the codes. They then independently coded four additional interviews before meeting to discuss and agree on codes once more. Finally, they independently coded five more interviews and met to discuss any disagreements or discrepancies. During each wave of coding checks, the coders discussed the inclusion and exclusion criteria, and how the meanings represented by the codes, as a form of data validation. The second coder acted as a critical friend, whereby the first author’s reflections, perceptions, and potential biases were discussed and critiqued. The remaining 50 % of the interviews, which were shorter in duration, were then single coded by the first author before zooming in and back out again to continue checking for any biases or preconceptions.

The next phase involved searching for broad patterns among the meanings and structuring categories, with a particular focus on those relating to participants’ experience of and within the group program, and its elements. The emerging categories were modified using the constant-comparison approach, where researchers compared newly uncovered and pre-existing codes. These categories were then clustered into derived themes and sub-themes. Using triangulation. All themes and underlying interview extracts were discussed, reviewed, and refined by both coders, and triangulation of the data was achieved with the inclusion of 7 participant reviewers who confirmed the themes were justified interpretations of their transcripts. Finally, themes were allocated names which were reflective of the lived experiences, and example transcripts were chosen. No qualitative analysis software was used on the data, so that coders could remain closer to the data. See Table 1 for example of theme development process.

**Table 1:** Example of theme development process (‘coding tree’).

Quotes	Categories	Final theme
We could all, we were all in the same boat, we could all understand each other	Relating to other members of BISEP	BISEP as a social milieu
So, it was good, and so good to see somebody who knows what you are going through.	Relating to other members of BISEP	
Because if someone cuts across when I am talking it knocks me back every time. It did not happen that much in the group.	Interacting with survivors is different to non-ABI	
If you were a group of normal people, if you like for lack of a better word for it, you know you are going to have to put up with questions and… it’s more of a…. strain. Where you are not going to have that quite so much from people undergoing quite a lot of the same problems.	Interacting with survivors is different to non-ABI	
Being able to share our experiences, our feelings, and strategies was informative, really, very much so.	BISEP as a place to share	
I found that I could share in the group which I thought was quite an achievement in a big group to be able to share things that are quite personal	BISEP as a place to share	
It was good, one could talk a bit more, not just about the accident but talk about the family, and what we have been up to.	BISEP as a place to socialize	
The people that where there made it better, because we had a laugh.	BISEP as a place to socialize	

### Participants

In total, twenty participants with ABI were invited to take part (17 men, 3 women), after first being approached over the telephone approximately two-weeks following attendance of a group psych-education program at the North Wales Brain Injury Service (NWBIS), Betsi Cadwaladr University Health Board (BCUHB). These twenty participants were from three separate but consecutive waves of BISEP (*n =* 8, *n =* 7, *n =* 5). The average age of the group was 50 (*SD =* 10.24, range 26–67). The average time since injury was 7 years (*SD =* 7.54, range 9 months – 32 years). Seven participants had suffered a cerebrovascular accident (CVA), 11 a traumatic brain injury (TBI), one who had an ABI following encephalitis, and one who sustained a hypoxic brain injury. All participants agreed to take part, and nobody withdrew from the study. The over-representation of men in the sample is reflective of the neurorehabilitation environment within NWBIS and within rehabilitation more generally (Frost et al., 2013).

### Procedure

The study was granted Ethical Approval by BCUHB (224613) and the School of Psychology, Bangor University. Participants were invited to attend the 7-session psycho-education group intervention, the Brain Injury Solutions and Emotions Program (BISEP). The program was facilitated by the first author (LR), and an additional member of the clinical team at NWBIS. The co-facilitator was kept constant as much as was possible. Each session lasted two hours, with a 15 min break approximately half-way.

Participants from three separate waves of BISEP took part in face-to-face qualitative feedback sessions, approximately 2 weeks after completing the program. It has been suggested that a minimum of twelve participants are required for data saturation (Guest et al., 2006), however, in the present study, data-saturation was judged to have been achieved through constant-comparison and analysis of data until no new themes emerged (*N =* 20). Interviews were carried out in a quiet room at Bangor University or NWBIS, and in patients’ homes in cases where travel was difficult. Only the researcher and participant were present for the sessions. The interviews lasted approximately 20 min (*M =* 18.52, *SD =* 6.38), were audio-recorded, and transcribed *verbatim*. A semi-structured interview format with prompts was used, i.e., “What are your thoughts on the BISEP?,” “What did you value most?,” “Are there any aspects that could be improved?,” “Has the program helped you understand how to manage your emotions better- in what way?,” “Have you used things from the sessions in your day to day life?.” Session-by-session prompting was used to assist participants’ recall. A collaborative interview approach with scaffolding was used, to help participants develop narratives (Paterson and Scott-Findlay, 2002; Carlsson et al., 2007, for a review). The questions were selected to be as open as possible, and the interview protocol was pilot tested with the first two participants. No changes were made to the protocol following the pilot testing, and data included in the present study. For interview protocol and additional prompts, see Appendix.

### The intervention – the Brain Injury Solutions and Emotions Program (BISEP)

BISEP, like all such group programs, has general elements, such as increasing awareness and understanding of injury, and facilitating adjustment. Strategies and compensation methods are also offered to help with common difficulties (e.g., problem solving and executive function, memory, and fatigue). Uniquely, the program is designed to have a strong emphasis on emotion, ER, and promoting positivity, and these elements are thus threaded throughout the entire program.

BISEP consists of: (1) an introductory session, (2) a session on anatomy and mechanisms of injury, (3) a session on emotional changes, (4) emotion regulation, (5) problem solving, (6) memory, and finally (7) fatigue. Participants receive a workbook every session, which includes the content, skill building and group exercises, and discussion prompts.

In the ‘Emotion Regulation’ session, ER strategies from the Process Model (situation selection, situation modification, attentional deployment, and cognitive change^1^) are conceptualized as ones to use ‘before’ (situation selection), ‘during’ (situation modification, attentional deployment), and ‘after’ (cognitive change) an emotional event. The positive element is threaded throughout the *entire* program, and includes the ‘Three Good Things’ intervention from the field of Positive Psychology (Seligman et al., 2005), as a *daily* homework. This involves making a note of three things that go well each day with a short causal explanation.

### Researchers’ reflexivity

The research was led by the first author (LR), who also conducted the interviews. LR, a female PhD student, had previous experience of qualitative research with people with ABI. She also received further training by the third and fifth authors (RC and OT), who have decades experience of clinical research with ABI participants. LR and CS coded the data, and CS, a clinical neuropsychologist, had previous experience of conducting qualitative studies with survivors of ABI. As mentioned, CS acted as a critical friend through the coding process, ensuring that we stayed close to the data. Finally, research themes and interpretations were scrutinized by SB through collaborative reflexivity with the first author, to ensure methodological rigor and challenge any underlying assumptions. No theme changes were required as a result of this process.

An important consideration was that the interviews were conducted by the BISEP facilitator (LR). The participants were, therefore, familiar with the researcher, and she would have known all participants for a period of 12 weeks (from group intervention recruitment/invites and pre-discussions, to the end of the 7 week program). This may have influenced the methodological rigor. In particular, participants may not have disclosed negative comments or critiques, or exaggerated the positive elements. However, an existing bond may have facilitated a conversational style of interviewing, that is recommended for participants with brain injury (Paterson and Scott-Findlay, 2002). The participants were informed that all their experiences and feedback were important for the evaluation, including constructive comments. All effort was made to encourage participants to feel comfortable disclosing issues. We also mitigated this by having a second coder (CS) who was not part of the service or the groups, and the fourth author (SB) who was external to the project to scrutinize the themes. As part of bracketing, the first author reflected and noted any assumptions, current knowledge, biases, and expectations based on her experience of running the groups and her subject expertise, and reflected on these within supervision. All effort was made to step away from these, and to interview and analyze the data from the position of an ‘unknower’, e.g., by asking prompting questions based on what is said in the moment and not any context or knowledge.

## Results

**Figure 1:**
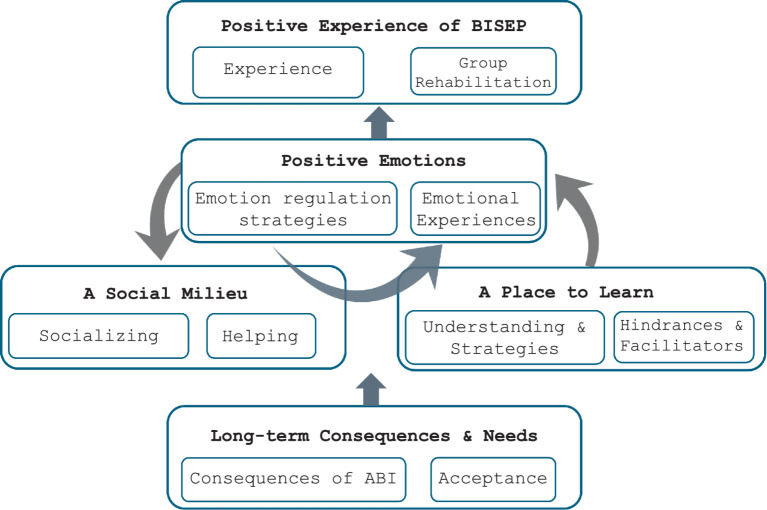
Visual representation of themes, and theme distribution.

Five themes of meaning were identified in the data, each consisting of two sub-themes. See Figure 1 for visual representation. The names associated with the quotes provided have been changed to protect participants’ identities.

### Theme one: long term consequences and needs of ABI survivors

This core theme (experienced by all participants but one) was related to the persistent nature of post-injury impairment, and how these result in a need for interventions such as the group program BISEP. The data show that in the early phases post-injury (9–24 months) or even in the chronic phase many decades following the injury, an ABI tends to affect every-day functioning in similar ways. Though the long-term consequences of ABI survivors were not directly related to the group program experience of environment, the presence of this experience in participants’ accounts was overwhelming, and highlighted their need for support (such as group intervention).

Two sub-themes were identified. The first related to the experiences of *direct* consequences of a brain injury, described by the majority of participants. This included cognitive impairments, fatigue, and difficulties with communication. Such difficulties resulted in some individuals having to *“take a step back”* from engaging in social activities. As described by Emyr:

*“… if someone cuts across when I am talking, it knocks me back every time […], so you tend to take a step back from everything because that happens. And like I said, when I am tired and when I have to really think to get the right words and things like that, it really tires me, so I take a step back.”*

And as described by Peredur:

*“The real problems are the ones I’m giving a good example of at the moment… That’s… communicating and… The main part of my brain, all the thinking bits, go on fairly normally… Unaffected… erm… and … so, I can sit in a chair and think about something and pretty effective at doing it, it’s only when making sense of it when trying to get it out.”*

The most common ABI consequence experienced by participants related to emotional changes and ER difficulties. Many participants reported experiencing low mood, or feeling *“so sad.”* A number also described how anxiety or *“panic”* made it difficult to engage in previously enjoyed leisure activities. Difficulty with *managing* emotions, or using maladaptive ways of coping, were also present in the data.

“*James: I’ve just, because I’m disabled, and I think about what I was like, before…*

*LR: So, when this sort of thing happens what do you do?*

*James: Go for a walk. Seems to be the only thing… I’ve got loads of things to do but I cannot do them. But bad things too, like having a drink. I had given up before this happened to me […] I do not drink much but I have started drinking again, it’s an escape really.”*

The second sub-theme related to the process of *acceptance* and adjusting to the long-term consequences described above (experienced by more than half of the participants). The dramatic change in functioning and identity from pre-injury levels, challenged participants’ ways of living, and was a common theme from participants early after injury to the chronic phase. This adjustment was described as analogous to a ‘journey’. For Emyr, the stark contrast to his pre-injury function, was difficult for him to *“get over.”*

*“I always go back to the same place in my head. Thinking about years ago, and the type of work I did in a day. In the past, if someone had called me saying there was a job in Scotland I would have just gone straight away there. Those things I miss. Those are the things I cannot get over at this time. It’s all behind me.”*

And for Gethin:

*“I was angry last year, I was… angry… why did it happen to me to start with, and angry it was taking me so long to recover. But this year, I’ve had to accept, and I have accepted, that it is going to take time. And maybe never get back to the way I used to be anyway […] Any progress I look forward to it like.”*

Other participants were objectively further on in their time since injury, but seemed earlier in their process of acceptance. For example, Huw:

*“I cannot accept it. It’s… do not know… I could do without it. It stopped me in my tracks.”*

### Theme two: positive experience of participating in BISEP

A second core theme related to participants’ general positive experience of participating in the program, and various valued elements which contributed to this experience (mentioned by all participants). Group members’ accounts especially reflect the relevance of group programs within neurorehabilitation.

Two sub-themes were, again, identified. The first related to the *positive experiences* of BISEP and various elements of the program. Some group members’ experienced that it took *“time to get into it.”* As participants relaxed in each other’s company many reported that they began to both *“enjoy”* and *“benefit”* from it. BISEP was a place where they felt positive, *“safe,”* free of judgment, and a place where they could benefit in terms of recovery.

*Cai: “We were there in our safe zone, nobody could judge us on anything, and we had a great time.”*

For a smaller number of participants, BISEP was a place where they experienced a sense of “*achievement”* toward the activities or end-of-program party, or developed *“confidence.”*

*Bevan: “… you think you have achieved something because you have done your little bit, and everyone else has done their little bit, so you can buzz off eachother.”*

The positive overall experience of BISEP appeared to become an important part of their routine, and something to “*look forward to”* each week. Half of the group members referred to the role of the facilitators, and their personalities, as especially important for a positive BISEP experience, and *“encourages people to think in [a positive] way.”*

As Iolo describes:

*“I think the group, I’d give it like a ten out of ten – it’s fantastic. Really everything about it, the people who run it, the personalities, the warmth, the knowledge, the care, the empathy as well […] People really bonded over the humour, and it was all because the whole group was run in such a positive way on every level. […] There was always this positive- it’s just walking in that room each week was like walking into a warm, sunny day.”*

These positive experiences of BISEP stood in contrast to participants’ experiences of acute care, and highlight the smaller sub-theme (described by half of the participants), *The relevance of group rehabilitation programmes.* Many participants expressed negative experiences of acute care, and a disconnect when returning to adjust in their community. In the face of these negative reflections, participants emphasized the relevance of group rehabilitation programs such as BISEP. Emyr expressed that there is a need for more rehabilitation resources in *“Cymraeg”* [the Welsh language], in particular. Individuals expressed that there is a need for the program to *“continue,”* and participants would like more opportunities to take part in groups. Consider the case of Rhys:

*“Get another one sorted. Sort another one as soon as possible. Do anything. And even if a group is set up again, I would attend the meeting.”*

And Angharad:

*“I just think, well, we are so lucky to have it. I think it should be… we are though, honestly. You know how the NHS is, it struggles massively, and how lucky are we to be able to have something like that.”*

### Theme three: BISEP was a social milieu

The third major theme related to the therapeutic function of socializing with other ABI survivors, within the context of the group rehabilitation program. This was reported by all participants.

The first sub-theme related to the experience and value of *socializing.* Participants valued the opportunity to be among people that were *“in the same boat,”* and could, therefore, *“understand”* much of their experience. This opportunity to *“share”* with people who can *“relate,”* in a setting of mutual understanding, was of greater value to many participants than *any* other component of BISEP. This appeared to be *“reassuring”* and brought a sense of normality to the group. For one participant, however, comparison to others was a *difficult* part of relating to other survivors, and contributed to negative emotional experiences.

For Gethin, the biggest value of BISEP was sharing with people who could relate to the effects of brain injury:

*“Gethin: Exchanging stories with the other people really. Yeah, we all have something in common, that’s the main point. […] Especially from the invisible injury side. Because we can all relate to each other in that respect.”*

Similarly, for Cai:

*“LR: What did you value most?*

*Cai: Erm… I would probably say, what jumps out of my mind, would be sitting down with people who have been through brain trauma like I have, and looking at them and listening to their stories, I would not say comparing them to mine but thinking: ‘He’s going through exactly the same thing as I did, and about the same time as I did’. I might be years on from what they are going through now. I also think that erm…staying in touch with some of them characters and helping them, if they had a bad day, or if I had a bad day and* vice versa*. That camaraderie that you can sort of get from a group like that is invaluable.”*

BISEP opened the possibility for people to *“enjoy a social setting in a managed way.”* That is, the positive experience of interacting with members of BISEP stood in stark contrast to their experiences of people who do not have a brain injury. Some participants reported difficult experiences of interacting with people. As described by Morgan, *“People that do not know that you have cracked your head, they look at you and they treat you like you are a bit of a, I do not know, a bit stupid.”* Peredur and Emyr both experienced pressures to act *“normal”* from people in the ‘outside’, which were substantially reduced when interacting with people in BISEP:

*Emyr: “I could tell people ‘I’ve had a brain injury’, but it just is not the same as if someone had broken a finger or arm, it just does not ever go away. And people from outside think ‘Oh, he looks ok now, he must be better, he is better now’. But it is not like that… So, in the groups, you come across people who understand, and are the same.”*

A smaller sub-theme, reported by few of the participants, was the opportunity to *help* other group members through sharing their experiences. For these participants, being in a position where they could help somebody else resulted in a positive emotional response. Helping others, or being able to *“contribute”* to the group, appeared to serve a positive function.

For example, the case of Lewis:

*“I sort of tried to help, what I’ve learned to pass on to them […] It was good for me. Made me feel good, put it that way. I’d come home and I’d think ‘Yes I’ve helped somebody today’. Whatever shape or form it took, you have still done a good thing. And I like that. So, doing that for them, was good for me.”*

### Theme four: BISEP was a place to learn

A key theme, reported by all participants consisted of the *learning* which took place during BISEP, and the many dimensions of that learning process. Two sub-themes were identified. The first, *developing an understanding of ABI and learning strategies to manage difficulties*, was experienced by the majority of participants.

Participants’ narratives suggest that they experienced the content as *“very informative”* and *“relevant.”* For many, they experienced BISEP as a place to learn about *“how the brain works,”* and through developing an understanding of brain injury they became *“less confused”* about their situation.

*Angharad: “Even though I knew bits from [previous career in medicine], I did not know it for me. I had never put me in that brain.”*

Participants also discussed learning specific “*strategies”* to help manage difficulties. The patients especially valued suggestions about *“tools for every-day life,”* and that adopting strategies could make *“life easier.”* Not only did participants value the learning which came from the content and the facilitator, but participants were able to learn from each other. Survivors were seen as a source of help similar to that provided by professionals.

In the case of Aled:

*Aled: The information definitely was comprehensive and very informative. I’ve taken on board quite a few of the strategies. And the strategies I’ve picked up off different clients. We were able to learn off each other and take information off each other. There’s so many different things you have mentioned that I’ve implemented really to make life easier for yourself. And it certainly does make life easier, and much more comfortable within yourself doing different things, having adopted strategies.”*

The second sub-theme related to things that *hindered* or *facilitated* learning in BISEP, described by three-quarters of participants. ABI consequences, such as difficulty with memory, or concentrating, could act as barriers to learning or engaging during BISEP. Related to this, three participants stated that there was *“too much information”* in sessions for it all to be digested. However, some participants described *implicit* learning which they may not be able to explicitly recall on the spot, but if a situation were to arise, they might implement learned material *“automatically.”* Consider, for example, the case of Cai who experienced that: *“in every meeting, you learn something, you might learn something subconsciously or consciously.”*

Participants also suggested that certain existing elements of the program *facilitated* learning, and helped *compensate* for challenges with the learning process. One such element was the use of 3D models of the brain and skull during the session about anatomy, to enhance experiential learning. Another essential component of BISEP that half of the participants found helpful was the handouts/booklets that they received with each session. These acted as a transitional resource, so that participants could *“refer back to”* and *“refresh”* their memories. This resource also allowed learning to continue even after BISEP has finished. Finally, some participants suggested that they would like even *more* sessions to go through things further, or to have an opportunity to revisit.

For Arwyn:

“*As I’ve said before, I often refer to the notes I’ve been given, and I refer to the notes that I personally take to refresh my memory, and also to think in different ways. Because… sometimes things do not occur to you at that particular time, but if you appraise, and go over what you have learned, you are more likely to find something that will stimulate you or present a strategy or whatever. It’s an on-going thing really.”*

These experiences of learning through the BISEP program demonstrate how knowledge is experienced as powerful for emotional healing post-injury, yet participants require significant support through learning resources and memory aids.

### Theme five: BISEP promoted positive emotional experiences

The final theme related to the emotional changes experienced through participating in BISEP, described by all the group members. For many, the program was associated with enhanced positive emotions, fewer experiences of negative emotions, and increased ability to *“manage”* emotional difficulties in daily life.

Two-sub themes were identified. The first, *emotion regulation strategies,* related to the use of strategies to manage emotional difficulties in daily life. Of the ER strategies included in BISEP, thinking of “*positive things”* was the most commonly reported helpful strategy, which was related to the ‘Three Good Things’ activity. Making a note of good things each day got *“people to talk and focus on the good,”* and *“engaged positive thought.”* The important thing, however, was the positive effect of doing this activity on mood, which appeared to continue following the program. For many, doing this helped them to *“notice positive things”* after BISEP, and encouraged *“positive thinking.”*

Twelve participants discussed the benefits of using ER strategies based on the Process Model, conceptualized in BISEP as tools to use ‘Before’, ‘During’, and ‘After’. The data indicated that the in the moment (‘During’) strategies were especially useful for many participants. This mainly consisted of distracting activities such as *“going for a walk”* to improve low mood, or *“moving away”* from overwhelming or anxiety-inducing situations. The ‘Before’ strategies seemed particularly salient for the few people who discussed them. By thinking things through beforehand, they could *“foresee [their] situation and predict where it’s going.”* Finally, a small number of participants described the benefits of the ‘After’ strategy of reappraisal for *“turning a negative into a positive.”* The ‘stop’ element was especially important for five participants.

As described by Rhian, *“And with the S.O.S […] I would practice it. I say things to my mum that makes for a bad atmosphere, because I’ve got no filter. But now I try to stop and think. And that has made me think, if you do just stop and think, I can stop that bad atmosphere. And it’s stupid that it has taken me until now.”*

For Robin, the ‘stop’ allowed him to *“buy some time”* which helped him deal with difficult situations.

The second sub-theme related to the *emotional experiences* that were related to the generic elements, and the philosophical approach, of BISEP. Participating in the program appeared to have beneficial effects on attendees’ emotional well-being, with one reporting *“feeling better”* after BISEP. For some individuals, they felt *“less aggressive”* after the group, or *“less critical”* of themselves and their progress.

As described by Iolo:

*Ermm … It’s helped me to be more aware of my emotions. And it’s helped me try and work through those emotions […] Ermm… things like even feeling low or whatever. I’ve got the dog. I do something positive. Think of something positive, focus on the positive. If I’m feeling low, the group helped me to feel the positive, getting up in the morning and being alive. It’s so important. The group helped you to look at that, remember that, focus on that. […] So, when bad emotions come along hopefully the positive will pull you out. So, yeah in that way yeah, definitely. […] But, the shopping one is probably most important […] Since I took the advice on shopping: Go to the shop, at a quieter time, go to a quieter shop. That was the -… things went a lot smoother for me in the shop. I still had my panic moments where I’ve forgotten and… Once I panic, I forget and I just want to get out […] the important thing is I have reduced my chances of having problems by doing things, and adapting when I do things, how I do things. I actually did not do that one week. And I went to a shop at a busy time…Thinking I can do this now, I can do it. And I went in and I was totally overwhelmed […] And it was absolutely awful, Because I did not follow these things. So, my problems have not changed really. But my ability to manage the problems has changed.”*

In sum, through the implementation of ER tools, developed in the BISEP group, a transfer could be seen into activities of daily living. For these participants, managing emotional reactions to post-injury challenges was key, and opened up an availability in survivors to compromise for the benefit of managing these emotional reactions.

## Discussion

To our knowledge, the present study is the first to directly explore participants’ experiences of and within a group program which specifically addresses ER (among other topics common in group psycho-education programs), and the core components that were valued by attendees. Using a descriptive phenomenology philosophy, the present study focused on lived experience and participants’ worldview within the group rehabilitation space. The discussion presents the patterns of meaning derived from individual’s experiences.

### Long-term difficulties need long-term support

Participants’ experiences emphasize the chronic nature of ABI in the *long-term*, consistent with previous literature (Hoofien et al., 2001; Dikmen et al., 2003; Colantonio et al., 2004; Salas et al., 2018; Dams-O'Connor et al., 2023). The findings also suggest that participants frequently disengage, or take a *“step back,”* from leisure pursuits and socializing, as a means of coping with the long-term difficulties (Fleming et al., 2011; Kersey et al., 2019, for a review), and may indicate a potential mechanism to address social isolation in rehabilitation. A recurrent element in the interviews was that survivors were at various stages of adjusting to changes in identity, or are on a different part of the ‘journey’ of acceptance, similar to themes identified by Couchman et al. (2014), and in a meta-synthesis by Villa et al. (2021). Interestingly, emphasizing the metaphor of a ‘journey’ might, in itself, be a tool which has important clinical implications (Huang and Aaker, 2019).

The persistent nature of participants’ difficulties, sometimes decades after the injury, highlight the important role of long-term community rehabilitation services (Wade, 2003; Coetzer et al., 2018; Norman et al., 2023), and group programs, in assisting patients with the adjustment process (Lundqvist et al., 2010; Lexell et al., 2013). The findings also contribute to the idea that addressing how survivors experience their ‘new self’ in rehabilitation might generate more positive adjustment (Gracey et al., 2008; Carroll and Coetzer, 2011). Together, this theme highlights the need for continued support, for example in the form of group programs, in the longer term.

### Experiences of valued elements

Participants experienced socializing with, and relating to, other survivors appeared to be a powerful, therapeutic experience (c.f. Salas et al., 2018). Being able to *“share,”* and relate to the experiences of people *“in the same boat,”* was something which connected the group members, and facilitated a sense of unity or cohesion, similar to previous studies (Lexell et al., 2013; Couchman et al., 2014; Salas et al., 2018). Existing holistic approaches traditionally include some emphasis on social interaction within rehabilitation (Gracey et al., 2010). Douglas et al. (2015) describe the need to emphasize social and relational approaches in neurorehabilitation, and promoting a sense of meaning and belonging after ABI. Participants’ interviews extend this idea by highlighting the potential benefit of developing a social milieu in promoting positive emotional experiences and a sense of connectedness (i.e., the feeling that they are *“not alone”*).

Participants experienced BISEP as a platform for *learning* to take place, consistent with previous literature (Lexell et al., 2013; Couchman et al., 2014). Attendees valued learning tools, or *“strategies,”* that could be used in their daily lives to manage various difficulties (e.g., memory, fatigue). This contributes to a well-established literature on compensation strategies (Wilson, 2000; Tsaousides and Gordon, 2009). Participants especially appreciated the strategies that they *“picked up off different clients,”* and the data suggests that other survivors can be a source of help similar to that provided by professionals.

Some participants experienced the consequences of ABI as *barriers* to learning in the program (e.g., *“sometimes things did not sink in”*). Rehabilitation has many ways to address difficulties in learning and memory following brain injury (Wilson et al., 1994; Evans et al., 2000; Kessels and Haan, 2003; Fish and Brentnall, 2016). The data from the present study especially suggest that experiential learning is provides a more positive learning experience, and that providing survivors with handouts or booklets is particularly valued, and allows learning to continue following program completion.

The final perceived important elements were the ER strategies, and positive philosophy of BISEP. The participants especially valued the ‘Three Good Things’ activity to manage low mood, and promote positive thinking. Previous literature has also indicated how making a note of ‘good things’ can help promote positive feelings (Andrewes et al., 2014; Karagiorgou et al., 2018), further highlighting the relevance of this activity in neurorehabilitation (Evans, 2011). Further, due to its simplicity it may be an especially beneficial tool for people with ABI.

In the context of the strategies based upon the Process Model (Gross, 2013), the ‘During’ strategies were frequently experienced as effective for managing emotional difficulties in daily life. These ‘in the moment’ tools are based upon ‘situation modification’ (modifying the *external* environment to manage emotional responses) and ‘attentional deployment’ (changing the *internal* environment to more favorable thoughts). Participants’ experienced these as useful for improving low mood, and managing anxiety or overwhelming situations. This is consistent with evidence in neurologically healthy participants, indicating that attentional deployment may be useful for those low in cognitive resources (Lohani and Isaacowitz, 2014; Sheppes et al., 2014).

Additionally, some participants described the relevance of the ‘Before’ strategies: the forward-thinking approach of ‘situation selection’. Through *“analysing”* and predicting the potential difficulties of various situations, participants reported being better able to manage their difficulties in daily life. This provides further evidence that situation selection may be particularly effective for people who struggle to regulate their emotions (Webb et al., 2018).

The ‘After’ strategies (reappraisal in the Process Model), was only explicitly described by three participants. However, there is some overlap between thinking of the positives generally with ‘Three Good Things’, and using positive things to change the meaning of a situation (as in reappraisal). However, reappraisal is a cognitively effortful strategy; which research suggests is difficult for patients with ABI (Salas Riquelme et al., 2014; Rowlands et al., 2019). It is possible that this strategy remains difficult to use, even after training, and that ‘Three Good Things’ may be a simpler approach to promote positive thinking.

Participants reported positive emotional experiences and changes, that were related to *generic* elements of the program. This emphasizes the role that a group program, with a positive philosophical approach, can have in improving emotional well-being following brain injury. The present study’s findings provide further support that group rehabilitation programs can be a promising vehicle to promote adaptive ER and emotional well-being (Tornås et al., 2016; Tsaousides et al., 2017).

### Acceptability of BISEP and the role of Group Interventions

BISEP was perceived as a valuable, beneficial, and a positive experience, similar to previous studies of group programs (Lexell et al., 2013; Couchman et al., 2014). The narrative especially suggests that enjoying the sessions played a central role in engagement, and participants’ motivation to attend the program until it was completed (*“I’m sad to see it end”*). The positive experience of participating in BISEP allowed participants to *“benefit”* in terms of their recovery (similar to Lexell et al., 2013; Couchman et al., 2014). A final point which deserves attention is the facilitators*’* role in fostering the positive rehabilitation experience. This contributes to well-established qualitative evidence, which points to the therapeutic relationship as a potential mechanism to promote rehabilitation engagement (Bright et al., 2015; Lawton et al., 2016), and demonstrates that it is not only *what* clinicians do that is important, but *how* they do it (Kayes and McPherson, 2012; Bright et al., 2015; Bishop et al., 2019).

Survivors described the relevance of group programs, such as BISEP, for community rehabilitation services. The lack of information provided at the organizational level in acute services resulted in feelings of disconnect after returning home, something which has been reported elsewhere (Piccenna et al., 2016; Abrahamson et al., 2017; Graff et al., 2018). The limited level of information provided to participants highlights how community services broadly, and group programs specifically, can help with progression through the rehabilitation pathway.

### Limitations

The results of the present study are promising. A potential limitation, however, is that the interviewer (LR) was also the person who facilitated the program. The impact of this was minimized by emphasizing the potential benefits of constructive criticism to improving the program for future, and taking time at the beginning of the session to reassure and create a sense of safety and receptiveness to all feedback. The researcher also used bracketing to aim to reduce any preconceived ideas or biases, and the second coder was not part of the BISEP program. Further mitigation was sought through including author SB to scrutinize the themes, and making use of clinical and academic supervision to support the reflectivity and bracketing process.

Future research would benefit from having an interviewer who was not the facilitator of the program and not involved in the service or program development, and to conduct interviews at an additional time point to track changes over time. It would also be interesting to interview family members or care-givers, to obtain an additional perspective. These aspects were beyond the scope of the current research project. An additional limitation is that the individuals who completed BISEP *chose* to enroll on a group program. The results would then be less generalizable to all survivors of ABI. Future research may benefit from unpacking people’s motivations for group rehabilitation and how this relates to their experience.

### Clinical and theoretical implications

Participant experiences suggest several important clinical implications. (1) *Build long term:* The findings provide clear support of the role of ‘slow stream’ holistic rehabilitation services in the *long term,* to help survivors with issues related to adjustment, acceptance, and identity. Services may benefit from ensuring a longer term pathway of care, and the literature may benefit from further research into models of rehabilitation and its barriers and enablers, in the chronic phase (2) *A social space:* The data highlight the importance of group programs in forming a *“safe”* and relational space, where participants can experience the therapeutic benefits of socializing with similar others. Additionally, by placing the focus of rehabilitation programs in the relational space between people, it provides a platform to learn from their peers, in addition to the formal content. There has been a growing literature on social connection and its relevance for rehabilitation. Continued research, in particular through a lived experience lens, would benefit the field. (3) *Provide strategies:* Suggesting practical tools and strategies may be a promising approach in rehabilitation programs. This is not a new idea in neurorehabilitation, but further research may be warranted to explore the perceived and objective efficacy of various tools. (4) *Focus on positives:* The present study suggests that BISEP (and other group programs) are promising vehicles for promoting positive emotional experiences, and adaptive ER skills. Participants especially noted the relevance of the ‘Three Good Things’ activity, in addition to the underlying principle of ‘promoting a positive outlook’ across all sessions.

## Conclusion

The majority of rehabilitation research has focused on post-acute services and individualized treatment (e.g., Cicerone et al., 2011). The present findings suggest that group programs are not simply an expedient tool to save money or clinician time. Group interventions, such as BISEP, have perceived benefits through the powerful therapeutic effect of shared experience. Patients identified therapeutic gains which the clinician themselves could not provide, but only facilitate. Finally, traditional rehabilitation interventions have tended to focus on cognitive impairment. The emphasis on emotion regulation and optimism had benefits which are arguably more important in patients’ lives. In sum, rehabilitation services may benefit from placing an emphasis on group programs, continued treatment in the chronic phase, and simple, practical tools that are delivered optimistically.

## Data availability statement

The datasets presented in this article are not readily available, because service and patient consent was not given to sharing full transcripts. Requests to access data should be directed to lrowlands@arden.ac.uk.

## Ethics statement

The present study, involving humans, was approved by Bangor University Ethics Committee and NHS Betsi Cadwaladr University Health Board. The studies were conducted in accordance with the local legislation and institutional requirements. The participants provided their written informed consent to participate in this study.

## Author contributions

LR: Conceptualization, Data curation, Formal analysis, Investigation, Methodology, Project administration, Writing – original draft, Writing – review & editing. CS: Formal analysis, Methodology, Writing – review & editing. RC: Conceptualization, Project administration, Supervision, Writing – review & editing. SB: Formal analysis, Writing – review & editing. OT: Conceptualization, Project administration, Supervision, Writing – review & editing.

## Footnotes

^1^Response modulation was not included in BISEP, as this strategy involves the regulation of emotional expressions not experience *per se.* There is also evidence that this strategy can be used maladaptively (Gross and John, 2003), and describing when/how to use it in an adaptive way might be confusing for patients.

## Appendix A

Semi-structured interview protocol

The interviewee used the following questions and additional memory prompts to explore group members’ experience of BISEP. Scaffolding was used when necessary to assist patients in developing a narrative and expressing their ideas.

- What are your thoughts on BISEP?
- Content – Session by session reminder prompts
- ‘Three Good Things’ activity
- Party
- What did you value most?
- Are there any aspects that you think could be improved?
- Has the program helped you understand how to manage your emotions better? In
- what way?
- ‘Before’, ‘During’, and ‘After’ strategies o ‘Three Good Things’ activity
- Have you used things from the sessions in your day to day life?
- Recap of main ideas/strategies from each session
- Do you have any additional comments?
